# Co-Occurring Atomic Contacts for the Characterization of Protein Binding Hot Spots

**DOI:** 10.1371/journal.pone.0144486

**Published:** 2015-12-16

**Authors:** Qian Liu, Jing Ren, Jiangning Song, Jinyan Li

**Affiliations:** 1 Advanced Analytics Institute, University of Technology Sydney, Broadway, NSW 2007, Australia; 2 Department of Biochemistry and Molecular Biology, Faculty of Medicine, Monash University, Melbourne, VIC 3800, Australia; 3 Centre for Research in Intelligent Systems, Faculty of Information Technology, Monash University, Melbourne, VIC 3800, Australia; 4 Advanced Analytics Institute and Centre for Health Technologies, University of Technology Sydney, Broadway, NSW 2007, Australia; Koc University, TURKEY

## Abstract

A binding hot spot is a small area at a protein-protein interface that can make significant contribution to binding free energy. This work investigates the substantial contribution made by some special co-occurring atomic contacts at a binding hot spot. A co-occurring atomic contact is a pair of atomic contacts that are close to each other with no more than three covalent-bond steps. We found that two kinds of co-occurring atomic contacts can play an important part in the accurate prediction of binding hot spot residues. One is the co-occurrence of two nearby hydrogen bonds. For example, mutations of any residue in a hydrogen bond network consisting of multiple co-occurring hydrogen bonds could disrupt the interaction considerably. The other kind of co-occurring atomic contact is the co-occurrence of a hydrophobic carbon contact and a contact between a hydrophobic carbon atom and a *π* ring. In fact, this co-occurrence signifies the collective effect of hydrophobic contacts. We also found that the B-factor measurements of several specific groups of amino acids are useful for the prediction of hot spots. Taking the B-factor, individual atomic contacts and the co-occurring contacts as features, we developed a new prediction method and thoroughly assessed its performance via cross-validation and independent dataset test. The results show that our method achieves higher prediction performance than well-known methods such as Robetta, FoldX and Hotpoint. We conclude that these contact descriptors, in particular the novel co-occurring atomic contacts, can be used to facilitate accurate and interpretable characterization of protein binding hot spots.

## Introduction

Residues at a protein interface always exhibit an uneven free energy distribution for the interaction [1]. Mutations on the majority interfacial residues have little effect on the binding free energy, but a mutation of the other interfacial residues (a small fraction of the interface) can significantly decrease the binding strength. This small fraction of interfacial residues is called a binding hot spot [1, 2]. Binding hot spots are thus of critical importance for our understanding of how proteins bind and function. Using wet-lab experiments, binding hot spots can be determined by site-directed mutagenesis such as alanine scanning mutagenesis [3]. However, experimental methods are often expensive, time-consuming and labour-intensive, and cannot be applied to characterize potential binding hot spots in a large number of proteins in a high-throughput and cost-effective manner.

As an alternative approach, a variety of *in silico* methods have been proposed to characterize and predict binding hot spots. These methods can be categorized into three groups: molecular simulation-based, empirical knowledge-based or machine learning-based methods [4]. The molecular simulation-based methods mutate candidate residues into alanines *in silico* and take advantage of molecular dynamics to examine the effect of mutations on the binding free energy change (ΔΔ*G*) [5]. Molecular simulation-based methods can achieve relatively high prediction accuracy, but they are too slow to be applied for high-throughput screening. Empirical knowledge-based approaches utilize experts’ prior knowledge—important factors that contribute to protein binding, and calibrate the weights of these factors such as hydrogen bonds, the van der Waals terms and Coulomb electrostatics, in a linear function for estimating ΔΔ*G* after residue mutations. Popular approaches in this group include FoldX [6, 7], Robetta [8, 9] and CC/PBSA [10].

More recently, machine learning-based methods have been proposed to study the effect of mutations [10–13]. These methods usually employ a set of features, ranging from hundreds to thousands, related to a mutation. These features, such as conservation, accessible surface area (ASA), residue propensity, residue pairwise potentials, van der Waals potentials, solvation energy, hydrogen bonds and Coulomb electrostatics, are extracted from different levels of heterogeneous protein data including sequence, structure and molecular interaction. Based on such features, machine learning algorithms, such as support vector machine (SVM), decision tree [14, 15], random forest [16], probabilistic model [4, 17] or Bayesian Networks [18], are used to learn the relationship between the features and ΔΔ*G* scores of the hot spot residues. Although some of these machine learning-based methods used the features derived from protein sequences only [19, 20] and others used the information from protein tertiary structures [21], most of them used different combinations of features from quaternary structures [21–23]. All the existing methods are useful; however, improvement in prediction performance is still desirable. Meanwhile, more interpretable and novel knowledge of binding hot spots needs to be investigated.

In this work, we propose a new computational method to improve the performance on the prediction of ΔΔ*G* and binding hot spots (upon alanine mutations). Our method explores the distinction capability of four types of features. These include: (i) the mutated atomic contacts and (ii) cross-interface atomic contacts in the neighborhood of a mutation residue. The third type of features relate to the co-occurrence of different types of atomic contacts in the neighborhood of a mutation residue. To the best of our knowledge, the co-occurrence of atomic contacts has seldom been investigated previously, despite its critical role in the contact cooperation of different atoms. For example, the co-occurrence of atomic contacts could capture the contribution of long-range interactions to protein interfaces. A previous work has shown that the non-interacting surface (a residue-based representation of long-range interactions), especially polar and charged residues, plays an important role in binding affinity [24]. This literature work strengthens our idea of co-occurring atomic contacts. The fourth feature type is related to B factor, which is a useful measurement reflecting the atomic vibrational motion. Interfacial residues in protein binding complexes have been found to have lower B-factors compared to the rest of the tertiary structural surfaces [25]. In this work, all atomic contacts are integrated with normalized B factors.

Our method uses a machine learning algorithm, random forest (RF) [26], to match the relationship between these features and ΔΔ*G* scores. The RF model is evaluated using leave-complex-out cross-validation on the ASEdb database [27]. It is also tested on the independent datasets BID [28] and SKEMPI [11], and on complexes which are either incorrectly labeled or not included in the dataset ASEdb or BID. This RF model can derive important features that contribute significantly to the accurate prediction of protein binding hot spots. These interpretable features can improve our understanding of binding hot spots and protein binding. The source code of our method can be downloaded from https://sourceforge.net/projects/pprf/files/.

We note that both the method proposed in this work and the one in our previous work [29] used *β* atomic contacts for the prediction of binding hot spots. Although sharing some similar ideas, the new method has five key points of differences/improvement from the previous method. (i) This work investigates co-occurring atomic contacts, while the previous work [29] did not; (ii) This work conducts thorough analysis of important features, while the previous work [29] did not; (iii) In [29], water exclusion hypothesis was integrated, while in this work, B factor is used instead; (iv) The method in [29] is developed based on a Ridge regression idea, while the classification method here is trained using RF. RF is used because it is able to produce a ranked list of important features. More importantly, the RF method can outperform the Ridge regression method when trained on the same feature space in this work—Details are reported in Tables 3 and 4. (v) The method in [29] is useful for hot spot prediction in protein-protein binding interfaces and might not perform well for prediction of protein-peptide binding (possibly due to the water exclusion effect); however, the method presented in this work is powerful even when tested on a dataset containing both protein-protein binding and protein-peptide binding. The study of protein-peptide binding is also important as it is involved in a wide range of biological processes and the small interfaces are attractive for therapeutic targets.

## Materials and Methods

### Dataset

#### The training dataset

The training dataset in this work contains 20 protein complexes, most of which are collected from the ASEdb database [27], denoted herein as the ASEdb dataset. Interfacial mutations for these complexes are defined by FoldX for the sake of making a fair performance comparison with FoldX and other existing works. All interfacial mutations are alanine mutations, and each of them is associated with a ΔΔ*G*. We note that the mutation of Gly to Ala is not considered because this kind of mutations might cause significant reconfiguration. The structures of protein complexes are also required to have available B factors in the Protein DataBank (PDB). Furthermore, an interacting protein pair in a complex must have no more than 40% sequence identity compared to interacting protein pairs in other complexes; otherwise, two protein complexes must have different mutations in similar proteins. The sequence identity in two given protein complexes (e.g., interacting pair A and B, and interacting pair C and D) is calculated using BLAST with the default setting for A and C, A and D, B and C, and B and D, denoted by *S*(*A*, *C*), *S*(*A*, *D*), *S*(*B*, *C*) and *S*(*B*, *D*) respectively. Two complexes are redundant if *S*(*A*, *C*) ≥ 40% and *S*(*B*, *D*) ≥ 40%, or *S*(*A*, *D*) ≥ 40% and *S*(*B*, *C*) ≥ 40%. Applying all the requirements above results in our ASEdb dataset with 366 alanine mutations. Of these, 79 are binding hot spot residues with ΔΔ*G* ≥ 2 kcal/mol.

#### The independent test datasets

The first independent test dataset has 19 protein complexes which are mainly retrieved from the BID database [28]. It is denoted as the BID dataset hereafter. A number of label errors in this dataset are corrected according to the original publications. Similar to the ASEdb dataset, interfacial mutations in the BID dataset are defined strictly according to the FoldX method. The mutations of Gly to Ala are not considered in this work, and the structures of protein complexes are required to have available B factors in PDB. All the 118 mutations in this BID dataset are annotated as ‘Strong’, ‘Intermediate’, ‘Weak’ or ‘Insignificant’. The 36 alanine mutations labeled as ‘Strong’ are considered as binding hot spot residues in this work.

The second independent test dataset is downloaded from SKEMPI [11]. Following the filtering step for constructing the ASEdb dataset, the PDB entries in the SKEMPI datset are also required to have low sequence identities with those in the ASEdb or BID datasets, or have significantly different mutations from similar proteins. The resultant SKEMPI dataset has 36 PDB entries with 232 alanine mutations. Of these mutations, 53 are hot spots with ΔΔ*G* greater than or equal to 2 kcal/mol. The requirements for the B factors in the PDB entries and the requirements for the interfacial mutations are set as the same as those for the ASEdb dataset.

### B factor

B factor, also known as temperature factor or Debye-Waller factor, measures the relative vibrational motion or the disorder of an atom in the protein crystal. It quantifies the displacement of an atomic position from its mean position in dynamic protein 3D structures and can be calculated using $B^{i}=8\pi^{2}U_{i}^{2}$, where $U_{i}^{2}$ is the mean square displacement of the atom *i*. As $U_{i}^{2}$ increases, B factor increases. A low B factor implies that the atom is in the well-ordered region of a structure, while a large B factor suggests a high flexibility of the atom. The distribution of B factor in different PDB structures varies greatly. We accordingly normalize the original B factor in this work. A normalized B factor is calculated using the following Eq (1).

$$\begin{array}{ccc} B_{norm}^{i} & = & \frac{B^{i}-\bar{B}}{\delta_{B}\times 1.645}\\ {\ddot{B}}_{norm}^{i} & = & max[min\left(B_{norm}^{i}-1,-2\right),0] \end{array}$$ (1)

where *B* ^*i*^ is the original B factor of the atom *i*, $\bar{B}$ and *δ*_*B*_ are the mean and standard deviation of the B factors of most atoms in the PDB of protein complexes, respectively. (After ranking the B factors of all atoms for a protein complex from the smallest to the largest values, 1% minimum atomic B factors are not used to eliminate possible errors with *B* ^*i*^ = 0, and 9% maximum atomic B factors are also excluded to remove outlier values. The two percentages are empirically determined.) Also, $B_{norm}^{i}$ is the normalized B factor of the atom *i*. The value 1.645 is a typical threshold under a standard normal distribution, indicating the 0.05 probability of a value outside [−1.645, 1.645] for each of the two tails. *min* means the minimum of two values, while *max* denotes the maximum. The first equation in Eq (1) is used to normalize and scale the 90% confidence interval of the B factor to [-1, 1]. The second equation in Eq (1) is used to set any value outside the 90% confidence interval to either -2 or 0, whichever is closer.

#### B factor-based vector

Two kinds of B factor-based features are used to describe each mutation. One is the averaged $B_{norm}^{i}$ for all mutated atoms, denoted by $B_{avg}^{r}$. The other is the difference (denoted by $B_{dif}^{r}$) of $B_{avg}^{r}$ and the averaged $B_{norm}^{i}$ for the backbone N and C atoms. The 20 standard amino acids are categorized into four groups: the first group contains ILE, VAL, LEU, MET, ALA and GLY, and the second group contains CYS, THR, SER, PRO, HIS, GLN and ASN. The charged residues, GLU, ASP, LYS and ARG, fall into the third group, while the remaining residues, *i.e.*, PHE, TRP and TYR, comprise the fourth group. Therefore, the B factor-based feature vector has 8 elements, where $B_{avg}^{r}$ are in (*p* ∗ 2)-th positions and $B_{dif}^{r}$ in (*p* ∗ 2 + 1)-th positions, and *p* is the amino acid group number to which a mutated residue belongs.

For comparison, two well-known Δ*ASA* (the change of the accessible surface area upon protein complexation) features are also used. One is the logarithm of Δ*ASA* of the mutated residues and the other is $\frac{\Delta ASA_{i}}{ASA_{i}}$, where *ASA*_*i*_ is the accessible surface area in proteins without binding partners.

### Atomic contact graph for an interface

Atomic contacts and their co-occurrence are intensively used for the prediction of protein binding hot spots in this work. Before defining the atomic contact features for a mutation residue, we illustrate how to generate an atomic contact graph for a protein binding interface. This process of generating contact graphs is similar to the process in our previous work [29], and is briefly discussed below for easy reference.

Given a protein complex *p*, we first identify interfacial atoms which have *β* contacts with their interaction partners. *β* contact is a new definition of atomic contacts [30] which requires that there are no other atoms interrupting the contact and assumes that two atoms should have enough direct contact area to form an important interaction. For this purpose, this contact definition requires two thresholds. One is a spatial distance threshold *T*_*d*_ of the contact between atom *i* and atom *j*. The other is ∠*β*, defining a forbidden region *fr* of the contact between *i* and *j*. *fr* is required to cover no other atoms. In this work, ∠*β* = 85, and *T*_*d*_ = 1.25 × (*vdw*_*i*_ + *vdw*_*j*_) where vdw_*_ is the van der Waals radius of the atom ∗ defined by [31]. Since there is no gold standard to determine the optimal thresholds for *T*_*d*_ and ∠*β*, the two thresholds are determined empirically. Previous works indicate that the number of atomic *β* contacts in protein binding interfaces only account for a small fraction of the number of distance-based contacts and less than half the number of contacts in the Voronoi diagrams [30]. More importantly, the use of *β* contacts has been demonstrated to be capable of achieving a better prediction performance in distinguishing false binding of crystal packing from homodimers [30], predicting binding hot spots and the change of binding free energy after mutations [29], and estimating protein-ligand binding affinity [32].

We identify all supporting atoms which have *β* contacts with any of the interfacial atoms, and then identify all covalently-bonded nearby atoms of all supporting atoms and of interfacial atoms as the neighborhood atoms if the nearby atoms are not backbone atoms. The covalently-bonded nearby atoms of a given atom *i* are those atoms within 3 covalent-bond steps of *i*. For example, given a chain of covalent bonds *i* − *j* − *k* − *l* − *m*, where − indicates a covalent bond. From *i*, the covalently-bonded step is 0 to *i*, 1 to *j*, 2 to *k*, 3 to *l*, and 4 to *m*, respectively. Thus, *i*, *j*, *k* and *l* are covalently-bonded nearby atoms of the atom *i*, while *m* is not.

All contacts involving neighborhood atoms (including supporting and interfacial atoms) are composed of an atomic contact graph for *p*. These contacts are used to extract atomic contact features.

A water molecule in PDB is considered as a part of an atomic contact graph if its accessible surface area is less than 1 Å^2^, and it has at least 3 *β* contacts with hydrogen bond donors (such as a nitrogen atom) and/or hydrogen bond acceptors (such as an oxygen atom). However, the contacts between any two water molecules are not considered.

### Atomic contact vectors for a mutation

We design three kinds of atomic contact vectors to describe a mutation. To generate these atomic contact vectors, we categorize all atoms of the twenty standard amino acids into 10 groups, as listed in Table A in S1 File. We then group all pairs of atomic contacts into 14 types (see Table B in S1 File). In particular, we also take into consideration *π* rings in PHE, TYR, TRP and HIS. In this work, each aromatic ring is represented by two pseudo atoms whose positions are just above the center of the ring and at about 0.5 *Å* spatial distance from the center. The atomic types of the pseudo atoms are also shown in Table A in S1 File. Based on these types of atomic pairs, three kinds of atomic contact vectors of a mutation are calculated as follows.

#### Mutated atomic contacts

Given a mutation, an atomic contact vector with 14 elements is used to describe the mutated atomic contacts. The mutated atomic contacts of a mutation are those contacts involving any of its mutated atoms. For each mutation, the value of an element *k* in this vector is calculated using $\sum ({\ddot{B}}_{norm}^{i}+{\ddot{B}}_{norm}^{j})/2$ for all contacts between *i* and *j* which belong to the *k*th group in Table B in S1 File.

#### Cross-interface atomic contacts in the neighborhood

Another atomic contact vector with 14 elements is also used to characterize the cross-interface atomic contacts that involve any of its neighborhood atoms (including mutated atoms and their supporting atoms) of a mutation. The element values of this vector are calculated in the same way as the vector of mutated atomic contacts.

#### The co-occurrence of atomic contacts in the neighborhood

The third vector is particularly used to represent the co-occurrence of atomic contacts in the neighborhood of a mutation. To the best of our knowledge, this is the first study to use contact co-occurrence to dissect protein binding hot spots. In this work, two atomic contacts, formed between atoms *i* and *j* or between *i*′ and *j*′, are considered to co-occur if *i*′ is *i*’s covalently-bonded nearby atom or *j*’s covalently-bonded nearby atom, or *j*′ is *i*’s covalently-bonded nearby atom or *j*’s covalently-bonded nearby atom. The contact co-occurrence can be used to illustrate the cooperation between atomic contacts. Given a mutation, the co-occurrence of mutated atomic contacts and their co-occurring contacts is represented by a vector which has 105(= 14 × 15/2) elements for all possible co-occurring pairs of the 14 types of atomic contacts. Given all co-occurring atomic contacts of *c* between the atoms *i* and *j*, and *c*′ between *i*′ and *j*′, assume that the atomic contact type of *c* is *t*_*c*_ and that of *c*′ is *t*_*c*′_; then, the value of the element (*t*_*c*_, *t*_*c*′_) is calculated by $\sum \left[\left({\ddot{B}}_{norm}^{i}+{\ddot{B}}_{norm}^{j}\right)/2+\left({\ddot{B}}_{norm}^{i^{\prime }}+{\ddot{B}}_{norm}^{j^{\prime }}\right)/2\right]$.

### Random forest learning model

Integrating all the aforementioned features, each mutation is represented by a feature set with 143 (= 10 + 14 + 14 + 105) elements to associate with its ΔΔ*G*. The relationship between the features of each mutation and its corresponding ΔΔ*G* is learned by a machine learning algorithm, random forest (RF) [26] as implemented in the randomForest package. This learning method is termed ppRF. In ppRF, only those features which each have more than 3 non-zero values in all mutations are used. In the randomForest learning process, 500 trees are built and every terminal node is required to contain at least three mutations.

Using randomForest, the importance score of each feature is also produced according to the difference in the performance before and after the permutation of the values of the feature. Note that after the permutation, the relation between the feature and the ΔΔ*G* becomes random. A feature with a larger importance score is generally considered to be more important in the learning process. Thus, the importance score can be used to assess a feature quantitatively. For the atomic contacts and the co-occurring contacts, especially, their unique contribution to the prediction of protein binding hot spots can be properly assessed.

### Evaluation measures

Our method ppRF and many existing methods generate a predicted ΔΔ*G* (ΔΔ*G*_*p*_ for short) for each mutation. We assess the performance of the methods by comparing this predicted value with the observed ΔΔ*G* value. To test the performance of ppRF on the independent BID dataset, a threshold (*T*_*hs*_) of the predicted ΔΔ*G* is used to define hot spot residues. *T*_*hs*_ = 1.5 kcal/mol is used under the assumption that ppRF underestimates ΔΔ*G*.

The prediction performance for binding hot spots is then evaluated by using the metrics in the following Eq (2).

$$\begin{array}{ccc} precision & = & \frac{TP}{TP+FP}\\ recall & = & \frac{TP}{TP+FN}\\ F1 & = & \frac{2\times precision\times recall}{precision+recall}\\ accuracy & = & \frac{TP+TN}{TP+FN+TN+FP}\\ specificity & = & \frac{TN}{TN+FP} \end{array}$$ (2)

where binding hot spot residues are considered as the true cases and non-hot spot residues as the false cases; TP, FP, TN and FN represent true positives, false positives, true negatives and false negatives respectively. *precision* is the number of correct hot spot predictions divided by the number of positive predictions, *recall* is the fraction of correct hot spot predictions over all hot spot residues, and *specificity* is the fraction of correct non-hot spot predictions over all non-hot spot residues. These measures are commonly used in [15, 22, 33] with the same definitions.

## Results

### Prediction performance

#### Test on three protein complexes to demonstrate that whether a residue becomes a hot spot residue is closely dependent on its binding partner

**Fig 1:**
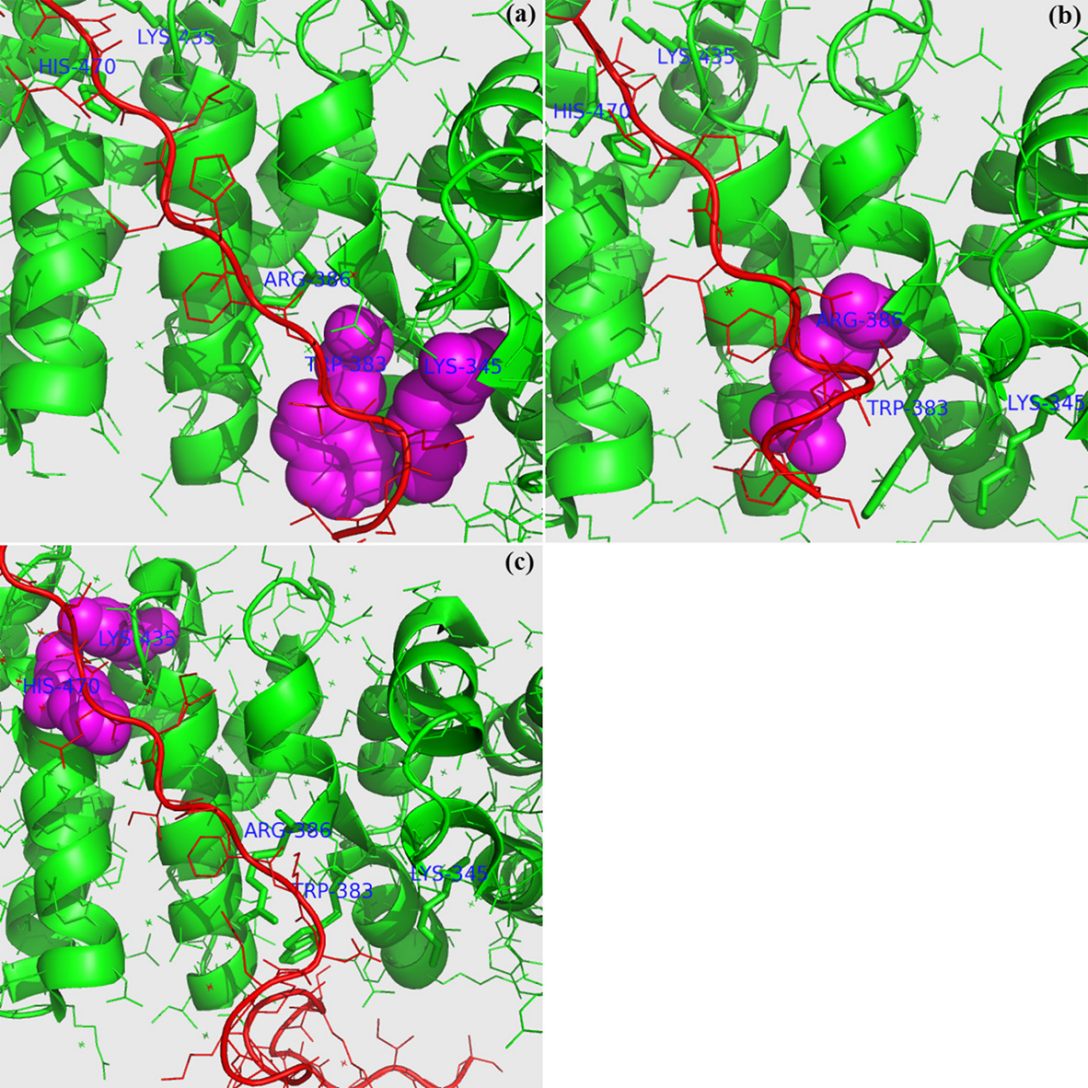
The binding hot spots [35] (in magenta) unique to 1TH1 (Chain A in green and Chain C in red in (a)), 1JPP (Chain A in green and Chain C in red in (b)) and 3OUX (Chain A in green and Chain B in red in (c)). There is an overlapping common area to the interfaces of these protein complexes; the chains in green are the same, but the binding partner proteins in red are different. The binding hot spots in Chain A (Lys-345 and Trp-383 in (a), Arg-386 in (b), and Lys-435 and His-470 (c)) are all in a ‘spheres’ view.

A residue of a protein can become a hot spot residue when the protein binds with a right protein, while the same residue may not be a hot spot residue anymore even when the protein uses almost the same binding site to interact with other partners. Our work was applied to three protein complexes to understand this point and to evaluate the performance of ppRF, Robetta, KFC2 (KFC2a and KFC2b) and Hotpoint [34]. These complexes are used here for two reasons. One is that they were either incorrectly labeled, or not included in the ASEdb or BID dataset of previous works (i.e., fresh benchmark data). The second is that they serve as good examples to illustrate that the binding hot spots of the same protein are not the same when the protein binds with different partners (forming different quaternary structures). The hot spots in the three complexes are shown in Fig 1 where the proteins in green are the same. The protein in green is able to use the significantly overlapping surface to bind with the three different partner proteins in red (not at the same time); however, the mutations of those overlapping residues make completely different contributions to the three complexes. For example, as shown in Table 1, the mutation of His470 would have an insignificant influence on the binding in Fig 1(a) and 1(b), while the mutation would strongly damage the binding in Fig 1(c). In another example, the mutation of Trp383 is a hot spot residue in Fig 1(a), but it is a non-hot spot residue in Fig 1(b) and 1(c). This kind of example exists widely in real world situations. Developing computational predictors that are able to capture the differences between such complexes and then accurately predict hot spot residues for each complex is thus technically challenging but practically extremely useful.

**Table 1:** Binding hot spot residues unique to the three complexes in Fig 1. The numeric real numbers are predicted values of ΔΔ*G*(kcal/mol). The *Observed* rows provide the BID-labels in the previous work [35].p.: precision; r. recall.

		Trp383	Arg386	Lys435	His470	p.	r.	F1
*Observed*	1TH1	*Strong*	*Insignificant*	*Insignificant*	*Insignificant*			
	1JPP	*Intermediate*	*Strong*	*Intermediate*	*Insignificant*			
	3OUX	*Insignificant*	*Intermediate*	*Strong*	*Strong*			
Robetta	1TH1	1.22	1.00	1.60	0.50	0.40	0.50	0.44
	1JPP	0.93	0.50	0.50	0.47			
	3OUX	2.58	2.38	1.69	3.05			
Hotpoint	1TH1	Hot Spot	Non-hot spot	Non-hot spot	Hot Spot	0.4	0.5	0.44
	1JPP	Non-hot spot	Non-hot spot	Non-hot spot	Hot Spot			
	3OUX	Hot Spot	Non-hot spot	Non-hot spot	Hot Spot			
ppRF	1TH1	1.06	2.61	1.76	2.40	0.38	0.75	0.50
	1JPP	0.325	1.65	1.12	0.99			
	3OUX	1.87	2.38	2.65	3.90			
KFC2a	1TH1	Non-hot spot	Hot Spot	Non-hot spot	Hot Spot	0.33	0.50	0.40
	1JPP	Non-hot spot	Hot Spot	Non-hot spot	Non-hot spot			
	3OUX	Hot Spot	Hot Spot	Non-hot spot	Hot Spot			
KFC2b	1TH1	Non-hot spot	Hot Spot	Non-hot spot	Non-hot spot	0.25	0.25	0.25
	1JPP	Non-hot spot	Hot Spot	Non-hot spot	Non-hot spot			
	3OUX	Hot Spot	Hot Spot	Non-hot spot	Non-hot spot			

We use four different methods to predict which of Trp383, Arg386, Lys435 and His470 are hot spot residues for the complexes shown in Fig 1. As an example, a correct prediction (ground truth) for His470 is that: this residue is not a hot spot residue in either 1TH1 or in 1JPP, but it is a hot spot residue in 3OUX (Please see Table 1). The prediction results are shown in Table 1. It can be seen that our method ppRF outperforms all other methods. It is not surprising that the prediction results of Hotpoint, KFC2a and KFC2b are almost the same for the three complexes. This is because these methods rely heavily on residue-level features, such as ASA, to make predictions and thus are incapable of capturing the change in atomic contacts in different complexes. In contrast, ppRF and Robetta are able to produce varying scores and ranking for the same mutations in the proteins of the three different complexes. For instance, ppRF produced the highest score for the mutation of Lys435 in 3OUX, because Lys435 has an ‘Insignificant’ contribution to the binding in 1TH1 and an ‘Intermediate’ contribution to the binding in 1JPP, but a ‘Strong’ contribution to the binding in 3OUX (Table 1). Also, ppRF labels more residues as hot spots on these three protein complexes than the other methods; thus it has a higher negative precision (the fraction of correct non-hot spot prediction over the number of predicted non-hot spots) and a worse specificity of non-hot spot prediction. Nevertheless, ppRF does not have worse specificity on the other datasets, as shown below.

Since several *in silico* methods have been proposed to predict ΔΔ*G* and binding hot spots using only protein sequences [19, 20] or protein tertiary structure information [21], our evaluation also suggests that more attention should be paid to the fact that binding hot spots are closely related to quaternary structures, if the quaternary structures or structural information are not available and the differences between protein complexes cannot be uncovered. Accordingly, a ground-truth dataset used in sequence-based or tertiary-structure-based hot spot prediction should be carefully constructed by considering the different mutational effects of the same residues at different complexes.

#### Leave-complex-out cross-validation performance of ppRF on the ASEdb dataset

We also evaluated several methods on the ASEdb datasets, including FoldX, Robetta, KFC and ppRF. The result is shown in Table 2, suggesting that ppRF outperforms all other methods with the highest F1 value of 0.570. Note that the performance of ppRF is obtained using rigorous leave-complex-out cross-validation tests. That is, all the hot spots in one complex are retained for testing, while hot spots in other complexes are used to train ppRF. The other methods are trained on a subset of our ASEdb dataset and published with a web server or executable program. They could not be re-trained under this cross-validation. Their performance is calculated using our ASEdb dataset as the input to their web servers or the local executables, generally leading to better performance than the predictor under cross-validation. In other words, the other methods under real cross-validation usually demonstrate worse performance than those in Table 2. Despite these aspects, ppRF still achieves better performance under strict real cross-validation than the other methods in Table 2.

**Table 2:** Performance comparison between FoldX, Robetta, KFC2 and ppRF on the ASEdb dataset. The performance of ppRF is evaluated using leave-complex-out cross-validation. FoldX, Robetta and ppRF are able to produce numerical values of ΔΔ*G*_*p*_. A predicted binding hot spot for these three methods is defined using ΔΔ*G*_*p*_ ≥1.5.

Method	Precision	Recall	F1	Accuracy	Specificity
FoldX	0.354	0.679	0.465	0.653	0.646
Robetta	0.438	0.548	0.487	0.743	0.799
KFC2a	0.427	0.772	0.550	0.727	0.731
KFC2b	0.511	0.582	0.544	0.790	0.847
ppRF	0.471	0.722	0.570	0.765	0.777

#### Performance evaluation on the independent datasets

The ppRF model is also tested on the independent datasets BID and SKEMPI. The prediction results on the BID dataset are shown in Table 3, and those on the SKEMPI dataset are presented in Table 4. The performance of our method is compared with those by FoldX, Robetta, KFC2 and HotPoint.

**Table 3:** Performance comparison between FoldX, Robetta, HotPoint, KFC2 and ppRF on the independent BID dataset, while all methods are trained using those mutations from the ASEdb dataset. FoldX, Robetta, Ridge and ppRF are able to produce numerical values of ΔΔ*G*_*p*_. The **HS** column indicates how to define a predicted binding hot spot given a predicted binding free energy ΔΔ*G*_*p*_: ≥2 suggests a predicted binding hot spot if its ΔΔ*G*_*p*_ ≥2, while ≥1.5 suggests a predicted binding hot spot if its ΔΔ*G*_*p*_ ≥1.5. ‘Ridge’ indicates the prediction results of Ridge regression on our feature space used for ppRF.

HS	Method	Precision	Recall	F1	Accuracy	Specificity
≥2	FoldX	0.514	0.528	0.521	0.703	0.780
	Robetta	0.577	0.417	0.484	0.729	0.866
	ppRF	0.692	0.500	0.581	0.780	0.902
HotPoint		0.552	0.444	0.492	0.720	0.841
KFC2a		0.560	0.778	0.651	0.746	0.732
KFC2b		0.697	0.639	0.667	0.805	0.787
≥1.5	FoldX	0.436	0.667	0.527	0.636	0.622
	Robetta	0.500	0.611	0.550	0.695	0.732
	Ridge	0.500	0.583	0.538	0.744	0.695
	ppRF	0.533	0.667	0.593	0.720	0.744

**Table 4:** Performance comparison between FoldX, Robetta, KFC2, HotPoint and ppRF on the SKEMPI dataset. FoldX, Robetta, Ridge and ppRF are able to produce numerical values of ΔΔ*G*_*p*_. A predicted binding hot spot for these three methods is defined using ΔΔ*G*_*p*_ ≥1.5. ‘Ridge’ indicates the prediction results of Ridge regression on our feature space used for ppRF, and ‘PCC’ denotes Pearson correlation coefficients between the predicted ΔΔ*G* and experimental ΔΔ*G*.

Method	Precision	Recall	F1	Accuracy	Specificity	PCC
FoldX	0.408	0.547	0.468	0.716	0.765	0.30
Robetta	0.472	0.642	0.544	0.754	0.788	0.39
KFC2a	0.412	0.755	0.533	0.698	0.682	
KFC2b	0.446	0.472	0.459	0.746	0.827	
HotPoint	0.339	0.377	0.357	0.690	0.782	
Ridge	0.382	0.491	0.430	0.765	0.703	
ppRF	0.492	0.585	0.534	0.767	0.821	0.52

It can be clearly seen from Table 3 that when *T*_*hs*_ = 2, our method ppRF achieves a F1 value of 0.581, which is almost 10 percent points higher than Robetta and 6 percent points higher than FoldX. In particular, ppRF achieves the precision value of 0.692, which is 18 percent points higher than FoldX, Robetta and HotPoint. The results indicate that around 70% of the predicted hot spots by our method are true binding hot spots, suggesting that ppRF is particularly useful when computational hot spots with higher accuracy are required with no available wet-lab evidence. When *T*_*hs*_ = 1.5 is used, the F1 values of all methods increase. Under this threshold, ppRF still achieves better performance than Robetta, FoldX and Ridge regression on the same feature space used by ppRF. In particular, the F1 value of HotPoint is 0.492, which is about 10 percent points lower than our method.

As shown in Table 4, our method ppRF achieves a competitive F1 value with Robetta and a considerably higher Pearson correlation coefficient than Robetta. Comparing with the other existing methods, ppRF outperforms again. Also on the SKEMPI dataset, we have investigated the change effect of *T*_*d*_ on the performance of the method. We found that our method has Pearson correlation coefficients from 0.492 to 0.54, and F1 scores from 0.48 to 0.564, when *T*_*d*_ changes from *T*_*d*_ = 1.1 × (*vdw*_*i*_ + *vdw*_*j*_) to *T*_*d*_ = 1.6 × (*vdw*_*i*_ + *vdw*_*j*_) where *vdw*_*i*_ and *vdw*_*j*_ are the van der Waals radii of two contacting atoms *i* and *j*. As there is no monotonic correspondence between the change of *T*_*d*_ and the change of performance, it is still an open question to find an optimal *T*_*d*_.

### Important atomic contacts

Important atomic contacts such as hydrogen bonds and salt bridges have previously been included in energy functions to estimate ΔΔ*G* [7, 8]. These atomic contacts are also important for hot spot prediction [7, 8, 36] and have been found to be closely related to hot spots [22]. More types of atomic contacts are worthy of intensive investigation to further our understanding of binding hot spots and protein binding. Using the randomForest model and the atomic contact graph representation, we analyzed all types of atomic contacts to examine (i) whether the important contacts revealed by the literature can be confirmed and (ii) whether new types of contacts critical to protein binding hot spots exist that were previously unknown.

**Fig 2:**
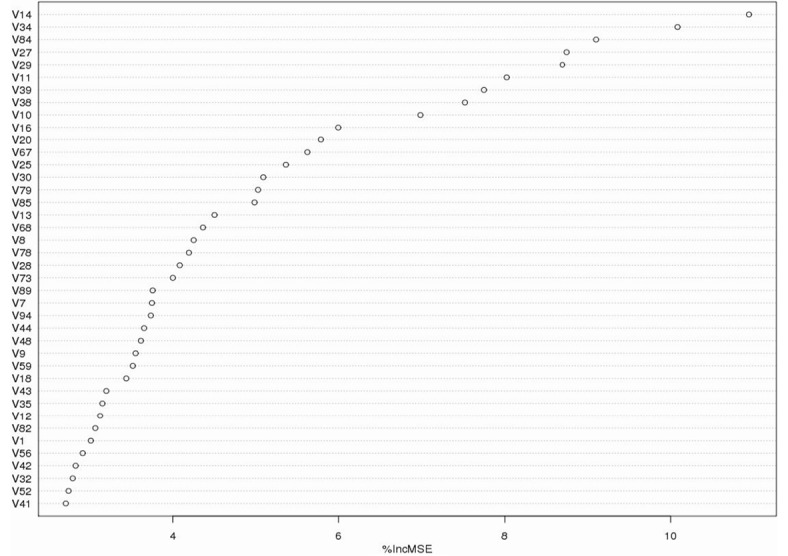
Top-ranking features useful for protein binding hot spot prediction by random forest. ‘%IncMSE’ indicates the increase of the mean standard error (MSE) after the permutation of the features. The definitions of the top 25 important features are listed in Table 5.

Each feature in the randomForest model is assigned an importance score. A larger score suggests that the feature provides irreplaceable knowledge in the prediction of binding hot spots. Trained on the ASEdb dataset, the top-ranked features and their importance scores are shown in Fig 2, whereas the meanings of the top 25 features are given in Table 5. We give examples as follows.

**Table 5:** Top 25 important features from Fig 2. R_*PCC*_ represents the Pearson correlation coefficient. As many features have tens or hundreds of zero values (refer to Figs 3, 4 and 5 for example), R_*PCC*_ itself is not able to produce a useful ranking here.

Features^1^	R_*PCC*_	R_*PCC*_ ^2^	^3^	Meaning^4^
V14	-0.315	-0.294	1	group 3: hydrophobic contacts between C_c^5^
V34	-0.277	-0.149	2	group 9: *π* involving contacts
V84	-0.273	-0.195	3	the co-occurrence of group 3 and group 9
V27	-0.525	-0.499	2	group 2: the contacts between C_c and oxygen/nitrogen
V29	-0.509	-0.501	2	group 4: C_on^6^ and oxygen/nitrogen
V11	-0.426	-0.425	1	group 0: hydrogen bond contacts
V39	-0.413	-0.390	3	the co-occurrence of group 0 and group 0
V38	-0.349	-0.473	2	group 13: the contacts between hydrogen-bond acceptors
V10	-0.054	-0.179		$B_{dif}^{r}$ of aromatic residues
V16	-0.300	-0.240	1	group 5: C_on and C_c
V20	-0.307	-0.408	1	group 9: *π* involving contacts
V67	-0.397	-0.326	3	the co-occurrence of group 2 and group 3
V25	-0.550	-0.530	2	group 0: hydrogen bond contacts
V30	-0.422	-0.356	2	group 5: C_on and C_c
V79	-0.351	-0.307	3	the co-occurrence of group 3 and group 4
V85	-0.235	-0.578	3	the co-occurrence of group 3 and group 10
V13	-0.399	-0.379	1	group 2: the contacts between C_c and oxygen/nitrogen
V68	-0.482	-0.501	3	the co-occurrence of group 2 and group 4
V8	-0.213	-0.439		$B_{dif}^{r}$ of charged residues
V78	-0.258	-0.230	3	the co-occurrence of group 3 and group 3
V28	-0.291	-0.213	2	group 3: hydrophobic contacts
V73	-0.295	-0.232	3	the co-occurrence of group 2 and group 9
V89	-0.234	-0.260	3	the co-occurrence of group 4 and group 4
V7	-0.313	-0.500		$B_{avg}^{r}$ of charged residues
V94	-0.208	-0.035	3	the co-occurrence of group 4 and group 9

^1^: features are in descending rank order according to the importance score generated by the random forest model in Fig 2.

^2^: R_*PCC*_ is calculated over mutations with non-zero feature values.

^3^: ‘1’ indicates mutated atomic contacts, ‘2’ indicates interfacial atomic contacts in the mutation neighborhood, while ‘3’ denotes the co-occurrence of atomic contacts.

^4^: ‘group X’ indicates the *X*th group of atomic contacts in Table B in S1 File.

^5^: C_c denotes carbon atoms which have no covalent bonds with oxygen/nitrogen atoms.

^6^: C_on denotes carbon atoms which have covalent bonds with oxygen or nitrogen atoms.

#### Top three features, all showing the hydrophobic effect

**Fig 3:**
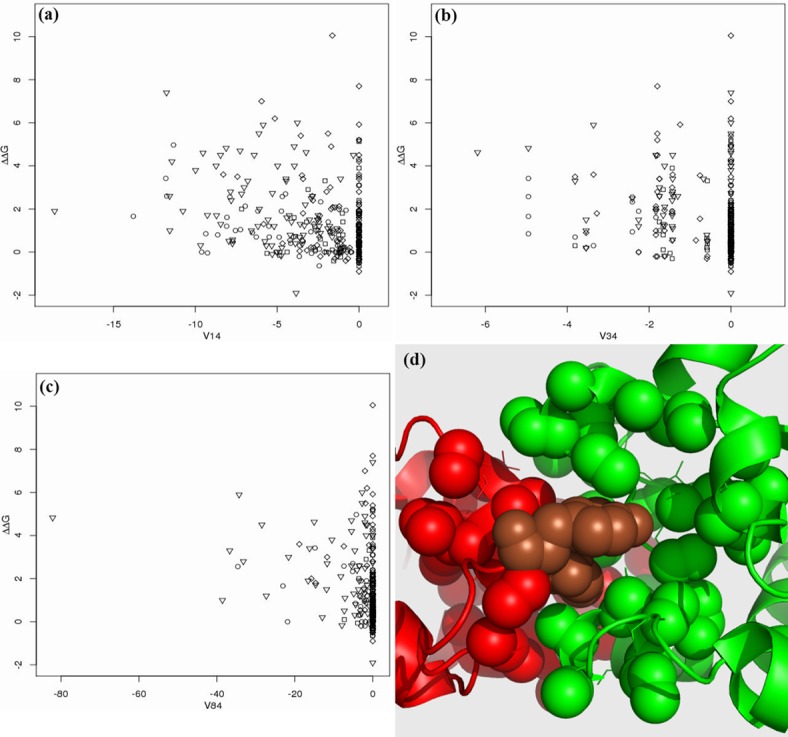
The distribution of top three features V14 (in (a)), V34 (in (b)) and V84 (in (c)). The definitions of V14, V34 and V84 are listed in Table 5. The importance of V14, V34 and V84 is ranked as 1^*st*^, 2^*nd*^ and 3^*rd*^, respectively, as shown in Fig 2, while the Pearson correlation coefficients of the three features are -0.315, -0.277 and -0.273, respectively, as shown in Table 5. The y-axes denote ΔΔ*G*. ∘: ILE, VAL, LEU, MET, ALA and GLY; ◻: CYS, THR, SER, PRO, HIS, GLN and ASN; ♢: GLU, ASP, LYS and ARG; ▿: PHE, TRP and TYR. (d) shows an example of the neighborhood of the two mutations (in brown): Tyr54 and Tyr55 of Chain A (in red) in 1BXI together with the partner protein (Chain B in green). All carbon atoms in the neighborhood which have no covalent bond with any oxygen or nitrogen are shown in ‘sphere’ view. The alanine mutation of Tyr54 has ΔΔ*G* = 4.83kcal/mol with the smallest value of V84, and that of Tyr55 has ΔΔ*G* = 4.63kcal/mol with the smallest value of V34.

As can be seen from Fig 2, the top first feature is the feature of mutated contacts (V14) among those carbon atoms (denoted by C_c for short) which have no covalent bonds with oxygen/nitrogen atoms. This ranking highlights the importance of the hydrophobic effect on protein binding hot spots. After the permutation of V14 in the randomForest learning process, MSE (mean standard error) increases by more than 10%. The distribution of V14 and ΔΔ*G* are shown in Fig 3(a) where the mutations with the smallest V14 values are almost hydrophobic residues (denoted by ‘∘’) in the first group and aromatic residues (denoted by ‘▿’) in the fourth group.

The top second feature in Fig 2 is related to the cross-interface contacts (V34) between *π* rings and C_c in the neighborhood of the mutations. This highlights the importance of both the hydrophobic effect and aromatic residues for protein binding hot spots. The permutation of V34 in the randomForest learning process leads to the increase of MSE by more than 10%. Its distribution together with ΔΔ*G* is shown in Fig 3(b) from which the same conclusion can be drawn as from V14.

The top third feature in Fig 2 is the co-occurrence (V84) of a contact in C_c and a contact between C_c and *π* rings. Although individual V14 and V34 both emphasize a hydrophobic effect on the prediction of binding hot spots, V84 still has a high ranking. Its permutation also leads to an increase of more than 9% MSE. This suggests that the co-occurrence of the two types of contacts represents a unique property of binding hot spots which is absent in individual V14 or V34. Thus, the clustering influence of hydrophobic contacts is of critical importance to protein binding.

An example of the hydrophobic effect is presented in Fig 3(d) where one mutation Tyr54 (ΔΔ*G* = 4.83kcal/mol) in Chain A of protein structure (PDB ID: 1BXI) has the smallest value of V34 in Fig 3(b), and the other mutations Tyr55 in Chain A has the smallest value of V84 in Fig 3(c). Fig 3(d) clearly shows that there are many C_c carbons around these two mutations, while the two residues also have *π* rings. Thus, in the neighborhood of the mutations, there are a large number of contacts between *π* rings and C_c, and their co-occurrence with the contacts in C_c, thereby illustrating why these two residues are important to protein binding. Interestingly, Tyr55 has a large ASA in the complex (*ASA* = 73.6 Å), but its ΔΔ*G* = 4.63kcal/mol is still quite high.

#### Hydrogen bonds and hydrogen bond network

**Fig 4:**
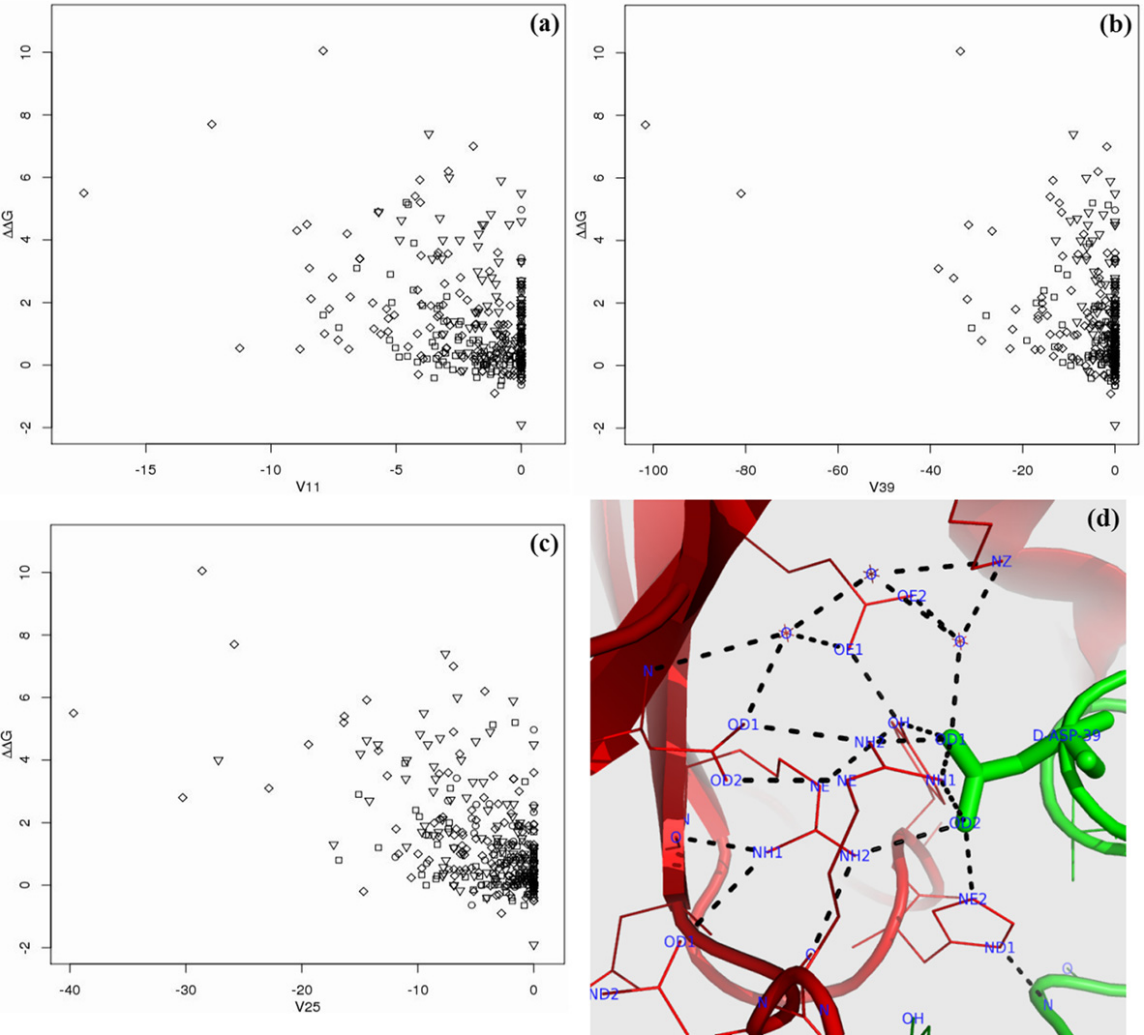
The distribution of three hydrogen-bond features V11 (in (a)), V39 (in (b)) and V25 (in (c)). The definitions of V11, V39 and V25 are listed in Table 5. The importance of V11, V39 and V25 is ranked as 6^*th*^, 7^*th*^ and 13^*th*^, respectively, as shown in Fig 2, while the Pearson correlation coefficients of the three features are -0.426, -0.413 and -0.550, respectively, as shown in Table 5. The y-axes denote ΔΔ*G*. ∘: ILE, VAL, LEU, MET, ALA and GLY; ◻: CYS, THR, SER, PRO, HIS, GLN and ASN; ♢: GLU, ASP, LYS and ARG; ▿: PHE, TRP and TYR. (d) shows an example of all hydrogen bonds in the neighborhood of the mutation (in ‘stick’ view): Asp39 of Chain D (in green) in 1BRS together with the partner protein (Chain A in red). The alanine mutation of Asp39 has ΔΔ*G* = 7.7kcal/mol and the smallest value of V39. The dashed lines represent potential hydrogen bonds whose acceptors and donors have spatial distance less than 3.5 Å.

Hydrogen bonds are widely considered to be an important factor for protein folding and binding. As can be seen from Fig 2, ppRF gives higher ranking for three kinds of hydrogen bond features: mutated hydrogen bonds (V11), cross-interfacial hydrogen bonds in the neighborhood of the mutations (V25), and the co-occurrence of hydrogen bonds in the neighborhood (V39). Their distribution of these features with ΔΔ*G* is shown in Fig 4. It is not surprising to see in Fig 4(a) that most of the mutations with the smallest feature (V11) values are from the third group (GLU, ASP, LYS and ARG). The neighborhood of these mutations also contains many hydrogen bonds, as shown in Fig 4(c). The co-occurrence of hydrogen bonds is shown in Fig 4(b) where the mutations with the smallest feature values are all hot spots. In fact, the co-occurrence of hydrogen bonds is a property of a hydrogen bond network. Fig 4(b) thus indicates the contribution of the hydrogen bond network to protein binding.

The importance of the hydrogen bond network is further illustrated in Fig 4(d) using an example of the mutation of Asp39 in Chain D of 1BRS (PDB ID). This mutation has ΔΔ*G* = 7.7kcal/mol with the smallest value of V39 in Fig 4(b). In Fig 4(d), many hydrogen bonds are localized in the neighborhood of the mutation, demonstrating how this network contributes to protein binding. In fact, there are seven other residues in this neighborhood network. Alanine mutation experiments have confirmed that five of the seven residues are true hot spot residues: Lys27 of Chain A with ΔΔ*G* = 5.4kcal/mol, Glu73 of Chain A with ΔΔ*G* = 2.8kcal/mol, Arg87 of Chain A with ΔΔ*G* = 5.5kcal/mol and His102 of Chain A with ΔΔ*G* = 6kcal/mol, and the Glu mutation of Arg83 of Chain A with ΔΔ*G* = 5.4kcal/mol. There is no experimental evidence to date for the other two residues. The extension of this neighborhood network contains two more alanine mutations: Tyr29 of Chain D with ΔΔ*G* = 3.4kcal/mol and Thr42 of Chain D with ΔΔ*G* = 1.8kcal/mol. This cluster of residues strongly supports the stability of cooperative hydrogen bonds in the network—the removal of any hydrogen bonds involving a residue would heavily affect the whole hydrogen bond network. It also provides evidence for the ‘hot region’ property of binding hot spots [37] (*binding sites—one side of the interfaces—might have several ‘hot regions’, locally tightly packed regions containing the clustered, networked, structurally conserved residues*) and for the ‘coupling’ theory [38] (*hot spot residues tend to couple a two-chain interface with higher local packing density*).

#### The various importance of B factor in the prediction of binding hot spots

Protein flexibility, as exemplified by B factor, is closely related to protein functions such as catalysis and allostery [39]. Deeply buried atoms in the core of the protein structure are often rigid and have low B factors [40], and interfacial residues in protein binding complexes also tend to have lower B-factors compared to the rest of the tertiary structural surface [25]. The importance of B factor in the prediction of binding hot spots has been investigated in a previous work [33] where the B factor based on the CA atoms was found to have an insignificant effect on hot spot prediction when used as an individual feature.

**Fig 5:**
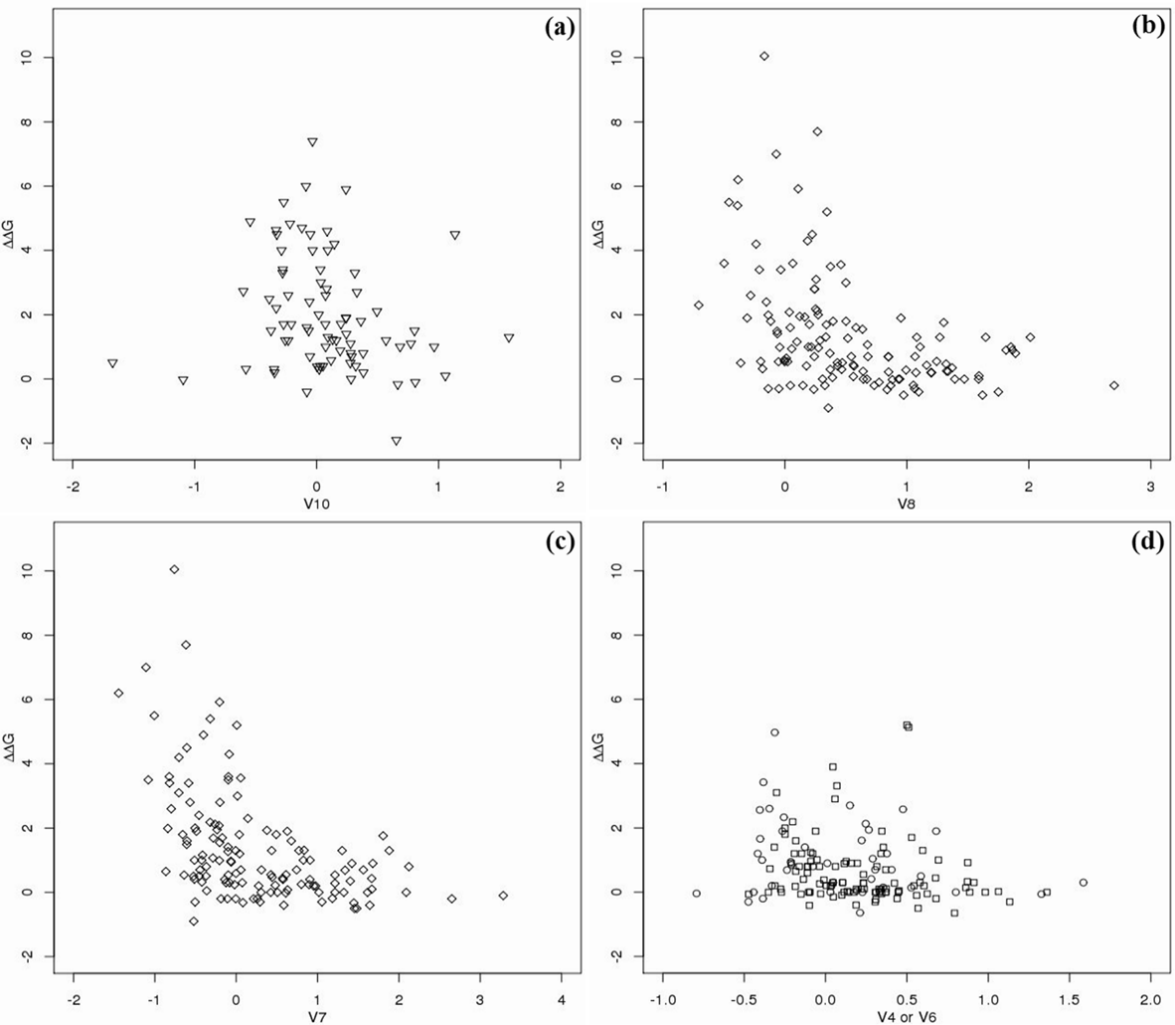
The distribution of $B_{dif}^{r}$ for the fourth amino acid group (in (a)), and for the third group (in (b)), and for the first and second groups (in (d)), and the distribution of $B_{avg}^{r}$ for the third group (in (c)). The y-axes denote ΔΔ*G*. ∘: ILE, VAL, LEU, MET, ALA and GLY; ◻: CYS, THR, SER, PRO, HIS, GLN and ASN; ♢: GLU, ASP, LYS and ARG; ▿: PHE, TRP and TYR. The importance of V10, V8 and V7 is ranked as 9^*th*^, 19^*th*^ and 24^*th*^, respectively, as shown in Fig 2, while the Pearson correlation coefficients of the three features are -0.054, -0.213 and -0.313, respectively, as shown in Table 5. V4 and V6 are not in the top 40 important features in randomForest.

In contrast to the previous approach, we concentrate on $B_{dif}^{r}$ and $B_{avg}^{r}$ rather than the B factor based on the CA atoms. It can be clearly seen from Fig 2 that $B_{dif}^{r}$ of the fourth group, and both $B_{dif}^{r}$ and $B_{avg}^{r}$ of the third group of amino acids make a significant contribution to the prediction of binding hot spots. To illustrate the various importance of the B factor for the four groups of amino acids, we also show the distribution of $B_{dif}^{r}$ for all four groups of amino acids and the distribution of $B_{avg}^{r}$ for the third group in Fig 5. It can be seen from Fig 5(b) and 5(c) that the larger values of $B_{avg}^{r}$ or $B_{dif}^{r}$ for the third group suggest fewer binding hot spots. Note that the third group contains GLU, ASP, LYS and ARG, which are all strongly charged residues. Thus, it is reasonable that charged residues lose motion freedom to contribute to protein binding. The $B_{dif}^{r}$ of the fourth group of the three aromatic residues (PHE, TRP and TYR) has a similar effect (Fig 5(a)) with the exception of two outliers of the smallest values. In contrast, the $B_{dif}^{r}$ of all other residues (Fig 5(d) for the first and the second groups of amino acids) contributes less to the prediction of protein binding hot spots. This might partly explain why the work in [33] drew the conclusion that the B factor of all types of amino acids in a same group contributed little to the accurate prediction of binding hot spots.

## Conclusions

We have proposed a new computational approach termed ppRF in this paper to characterize protein binding hot spots. Our method ppRF integrates the contributions from multiple informative features, including B factor, mutated atomic contacts, neighborhood cross-interface contacts and co-occurring neighborhood contacts. Assessed on independent test datasets and under cross-validation, ppRF achieves a significant improvement compared to competitive methods in the literature. ppRF is able to detect features which make a unique contribution to the prediction of binding hot spots. Some features have been investigated and widely used in existing energy prediction functions, such as hydrophobic contacts and hydrogen bonds. Some features have seldom been studied previously, such as the co-occurrence of hydrophobic contacts and *π* involving contacts, and hydrogen bond networks. In conclusion, this work not only presents a more accurate prediction method, but also provides important novel insights into the atomic-level rules of protein binding hot spots.

## Supporting Information

S1 File Supporting tables. The groups of atomic types and the groups of atomic pairs.
(PDF)
