# Supplementary material for: Co-Occurring Atomic Contacts for the Characterization of Protein Binding Hot Spots
Source: PLoS One. 2015 Dec 16;10(12):e0144486. doi: 10.1371/journal.pone.0144486 (PMC4684219; doi:10.1371/journal.pone.0144486)
Supplement: S1 File — The groups of atomic types and the groups of atomic pairs. (PDF) [file pone.0144486.s001.pdf]

Co-occurring atomic contacts for the characterization of protein binding hot spots—supplementary

Qian Liu[1], Jing Ren[1], Jiangning Song[2,3], Jinyan Li[4,*]
1 Advanced Analytics Institute, University of Technology Sydney, Broadway, NSW 2007, Australia
2 Department of Biochemistry and Molecular Biology, Faculty of Medicine, Monash University, Melbourne, VIC 3800, Australia
3 Centre for Research in Intelligent Systems, Faculty of Information Technology, Monash University, Melbourne, VIC 3800, Australia
4 Advanced Analytics Institute and Centre for Health Technologies, University of Technology Sydney, Broadway, NSW 2007, Australia
∗ E-mail: jinyan.li@uts.edu.au

Group		
id	name	Description
0	0.DA	atoms which can be hydrogen bond donor and acceptor
1	1.D	atoms which can be hydrogen bond donor only
2	2.A	atoms which can be hydrogen bond acceptor only
3	3.C	carbon atoms which have no covalent bonds with any nitrogen or oxygen
4	4.ARO	pseudo atoms representing aromatic ring in amino acids such as PHE, TYR, and TRP.
5	5.ARH	pseudo atoms representing aromatic ring with at least one non-carbon atom in amino acids such as HIS and TRP.
6	6.Cda	carbon atoms which have covalent bonds with both 1.D and 2.A.
7	7.Cd	carbon atoms which have covalent bonds with 1.D only.
8	8.Ca	carbon atoms which have covalent bonds with 2.A only.
9	9.HOH	oxygen atoms in water molecules

Table A. The group of atoms for 20 standard amino acids and water molecules.

	**0.DA**	**1.D**	**2.A**	**3.C**	**4.ARO**	**5.ARH**	**6.Cda**	**7.Cd**	**8.Ca**	**9.HOH**
**0.DA**	0	0	0	2	7	8	4	4	4	0
**1.D**	0	1	0	2	7	8	4	1	4	0
**2.A**	0	0	13	2	7	8	4	4	1	0
**3.C**	2	2	2	3	9	10	5	5	5	2
**4.ARO**	7	7	7	9	11	6	7	7	7	7
**5.ARH**	8	8	8	10	6	12	8	8	8	8
**6.Cda**	4	4	4	5	7	8	4	4	4	4
**7.Cd**	4	1	4	5	7	8	4	1	4	4
**8.Ca**	4	4	1	5	7	8	4	4	1	4
**9.HOH**	0	0	0	2	7	8	4	4	4	-1

Table B. The group of atomic pairs for the 10 types of atoms in Table A. The names in the first column and the first row represent the 10 types of atoms, and the other numbers represent the group id of atomic pairs. For example, group 0 represents potential hydrogen bonds. -1 states that this kind of atomic contacts are not considered.
